# The Impact of Accelerated Digitization on Patient Portal Use by Underprivileged Racial Minority Groups During COVID-19: Longitudinal Study

**DOI:** 10.2196/44981

**Published:** 2023-08-09

**Authors:** Feng Mai, Dong-Gil Ko, Zhe Shan, Dawei Zhang

**Affiliations:** 1 School of Business Stevens Institute of Technology Hoboken, NJ United States; 2 Department of Operations, Business Analytics, and Information Systems University of Cincinnati Cincinnati, OH United States; 3 Department of Information Systems and Analytics Miami University Oxford, OH United States; 4 Department of Decision and Technology Analytics Lehigh University Bethlehem, PA United States

**Keywords:** digital divide, race, health care, patient portal, mobile health, accelerated digitization, COVID-19, mobile phone

## Abstract

**Background:**

Prior research on the digital divide has documented substantial racial inequality in using web-based health resources. The recent COVID-19 pandemic led to accelerated mass digitization, raising alarms that underprivileged racial minority groups are left further behind. However, it is unclear to what extent the use of health information and communications technology by underprivileged racial minority groups is affected.

**Objective:**

We have considered the COVID-19 disruption as a rare exogenous shock and estimated the impact of the accelerated digitization on the quantity and variety of patient portal use. In this study, we aimed to answer the following 2 key research questions. Did patients alter their use of health information and communications technology owing to COVID-19–induced digital acceleration? Does the effect differ across racial lines?

**Methods:**

We used a longitudinal patient portal use data set gathered from a large urban academic medical center to explore the effect of accelerated digitization on the racial digital gap in health care. We limited the sample period of our study to 2 same periods (March 11 to August 30) in 2019 and 2020. Our final sample consisted of 25,612 patients belonging to 1 of the 3 racial groups: Black or African American (n=5157, 20.13%), Hispanic (n=253, 0.99%), and White (n=20,202, 78.88%) patients. We estimated the panel data regression using 3 different models: pooled ordinary least squares (OLS), random effect (RE), and fixed effect (FE).

**Results:**

Our study yielded 4 findings. First, we confirmed that the racial digital divide remains a significant issue for telehealth; underprivileged racial minority group patients had lower patient portal use than White patients before the pandemic (*Minority*: OLS, β=−.158; *P<*.001; RE, β=−.168; *P*<.001). Second, we found that the digital gap regarding patient portal use frequency between underprivileged racial minority groups and White patients is *shrinking* rather than widening after the COVID-19 pandemic started (*COVID_Period×Minority*: OLS, β=.028; *P=*.002; RE, β=.037; *P<*.001; FE, β=.043; *P<*.001). Third, the shrinking gap is foremost driven by access through mobile (vs desktop) devices (*COVID_Period×Minority*: web, β=−.020; *P=*.02; mobile, β=.037; *P<*.001). Finally, underprivileged racial minority groups expanded their use of a variety of portal functionalities faster than White patients during the pandemic (*COVID_Period×Minority* [for functionality]: OLS, β=−.004; *P<*.001; RE, β=−.004; *P<*.001; FE, β=−.003; *P=*.001).

**Conclusions:**

Using the COVID-19 pandemic as a natural experiment, we offer empirical evidence that accelerated digitization has shrunk the racial digital divide in telehealth, and the trend is mostly driven by mobile devices. These findings provide new insights into the digital behaviors among underprivileged racial minority groups during accelerated digitization. They also offer policy makers an opportunity to identify new strategies to help close the racial digital gap in the postpandemic world.

## Introduction

### Background

Health care services across the world have been severely disrupted by COVID-19. A confluence of factors, including overflowing of patients with COVID-19, shelter-in-place orders, and the general reluctance to visit medical facilities, has limited health care access for patients. Delay or avoidance of care during COVID-19 has grave consequences, such as increased morbidity and mortality rates associated with other health conditions [1]. According to the Centers for Disease Control and Prevention [2], telehealth, in both synchronous and asynchronous forms, can be a crucial remedy to the problem during the pandemic. Indeed, prior information systems (IS) research [3,4] and contemporary clinical evidence [5,6] suggest that telehealth can help address many challenges in providing health services during an outbreak and improve health outcomes for patients.

As health care increasingly moves over the web, the issue of the digital divide has been at the center of discussion. The digital divide refers to the inequality in the access and use of IT across demographic, racial, and geographic dimensions [7-9]. The US Census Bureau [10] highlights the access disparity across the racial dimension; Black or African Americans and Hispanic individuals are less likely to have broadband connections than White individuals. However, even when racial minority group patients have access to the internet and could register for accounts, they are still less likely to effectively *use* health care information and communications technologies (ICT) [11]. Amid the pandemic, many warn that the rapid shift to web-based health care services has widened such a divide, disproportionately hurting underprivileged racial minority groups [12]. If such concerns are legitimate, the digital divide not only threatens to undo the progress in the development of telehealth technology but also exacerbates preexisting income and racial inequalities in obtaining health care.

However, despite its toll, COVID-19 also presents a rare opportunity for *accelerated digitization*, a term used to describe the rapid adoption of digital technologies by individuals and organizations as a response to the pandemic (terms with similar meanings include *digital acceleration* or *accelerated digitalization*) [13-15]. A survey of company executives estimated that the pandemic has sped up their digital offerings by 4 to 7 years [15]. Web-based customer interaction was cited as one of the biggest factors, whereas the health care sector reported the most significant changes. Although these changes have been mostly out of necessity, because of the logistical advantages it brings during a time when almost everything has moved over to the web, the prospect of these benefits is motivating industry leaders to further accelerate their digital transformation journey [16]. It is thus plausible that the rapid digital transition also elicits long-term adaptive responses from patients, providing a nudge to those who have traditionally underutilized technologies [17]. As such, many nongovernmental organizations urge that accelerated digitization should be viewed as an opportunity to recognize and bridge the digital divide [18]. Despite the spotlight on this issue, there is limited direct evidence on the impact of accelerated digitization from the patients’ perspective. Did patients alter their use of health ICT because of COVID-19–induced digital acceleration? Does the effect differ across racial lines?

An inquiry into these questions is important for 2 reasons. First, much of the previous research on the digital divide at the individual level has been limited to survey data [9,19,20] or relies on a retrospective, normative approach [7]. In contrast, the COVID-19 pandemic offers a rare opportunity to use longitudinal, granular, and archival use data for examining how the digital divide naturally manifests itself as the pandemic runs its course. Such an empirical examination is crucial because “if the level of disparity was to grow or even if it was to stay the same while the economic importance of the disparity was to grow due to the increasing ubiquity of digitization—then it will exacerbate inequality” [21]. In other words, studying how underprivileged racial minority groups fared under the accelerated digitization caused by the COVID-19 pandemic provides a unique opportunity to examine the impact of this rapid transition on preexisting inequalities and assess whether the future of telehealth is prone to perpetuating or exacerbating these disparities.

Second, this study can inform initiative-taking intervention efforts aimed to bridge the racial digital divide. Various initiatives have been proposed at global and local levels including the distribution of low-cost or free devices, expansion of broadband network coverage, and subsidies for internet service plans [22,23]. However, there are limits to what blanket measures can achieve, especially when they do not consider specific health care use settings. Because benefiting from telehealth requires more than merely owning an account and having internet access, understanding how racial minority group patients use telehealth is vital for providing targeted support and making sound policy decisions.

In this study, we empirically evaluate whether COVID-19–induced accelerated digitization has widened the racial digital divide. We obtained a unique patient-level data set from a large academic medical center (AMC) in the United States; the data set contains patient portal use [24] and corresponding electronic health records (EHRs). It is important to note that portal use aligns with a broad definition of telehealth [2], that is, portal use includes multiple modalities including asynchronous and synchronous communication and remote transmission of health records. Our dependent variables were (1) the monthly frequency (sessions) of the portal use and (2) the variety of functionalities that the patients use. We compared the portal use between underprivileged racial minority groups and White patients before and after the COVID-19 pandemic started. To ensure that the COVID-19 pandemic had a similar degree of impact on patients in our sample, we controlled for a wide variety of factors in our regression analyses including age, gender, health conditions, income, number of office visits, education attainment, medical problems, and whether a patient had a positive COVID-19 test result and had been exposed to SARS-CoV-2. In addition, we conducted a propensity score–matching analysis to ensure that our results were not driven by the degree to which the patients were affected by the pandemic.

### Related Literature

#### Digital Divide

Our work is rooted in the digital divide literature. Dewan and Riggins [7] developed a framework that defines 3 levels of the digital divide: individual, organizational, and global. For each, there are 2 orders of effects: first-order effects where the focus is on the divide in *access* to technology and second-order effects on the inequality in the ability to *meaningfully use or fully benefit* from the technology among those who have access. Most of the prior IS research focused on the first-order effects. The recent rapid penetration of broadband and mobile internet use has contributed to shrinking the digital divide in access [25,26]. However, even when individuals do have equal access to digital technology, differences in skills can lead to digital inequalities [27,28]. Prior research has paid little attention to such second-order effects on the individual-level digital divide, and their findings are often inconsistent [8,9,19,29]. Following this stream of research, our study focuses on the second-order effects and seeks to understand the individual-level digital divide in patient portal use.

#### Racial Digital Divide in Health Care

Concerns about the digital divide in health care have risen, as both patients and providers are increasingly relying on the internet and digital technologies [30]. Over the past decade, medical providers have promoted the use of patient portals, which help patients access health care services and information such as their laboratory results, medical histories, appointment schedules, and prescriptions [31,32]. Portals also provide both synchronous and asynchronous electronic communication between patients and physicians. The literature attests to the benefits of e-visits and portal use in improving patient health [3,4]. Despite the widespread availability and proven benefits, a digital divide in portal use persists. The disparity is more acute across the racial dimension than across any other patient characteristics such as age, gender, or health status [33]; compared with White patients, underprivileged racial minority group patients significantly underutilize the patient portal and other digital health resources. Not only does such a digital divide pose a threat to ensuring that health ICT can benefit everyone equally, but it can also exacerbate the long-standing racial health disparity [34]. However, the racial digital divide in health care is rarely addressed in IS literature. Our study fills this gap when the issue has become more urgent with accelerated digitization.

#### Accelerated Digitization and Racial Digital Divide

Given that racial minority groups have historically underutilized telehealth, how does accelerated digitization alter their behavior and change the racial digital gap? Social cognitive theory (SCT) offers a theoretical lens to answer this question [9,35]. According to SCT, human behavior is the product of a dynamic interplay between 3 sets of influences: personal, behavioral, and environmental factors. In our context, personal factors (eg, race, age, self-efficacy, income, and health conditions); environmental conditions (eg, challenges and opportunities presented by accelerated digitization); and behavioral factors (eg, desktop vs mobile internet use) should collectively engender social cognitive influence on how patients use telehealth.

Following this line of reasoning, there are 2 sides to the argument regarding how the preexisting racial digital divide may shift. First, it is reasonable to believe that the racial digital divide will widen because of well-entrenched personal factors. One of the most important personal factors in SCT is self-efficacy, defined as beliefs about one’s ability to perform a specific task [36-38]. Prior literature suggests that self-efficacy is an important precursor to the adoption of new technologies, particularly in health care contexts such as mobile health (mHealth) apps [39] and telemedicine services [40]. White patients on average have higher self-efficacy because they are early adopters of patient portals and have more prior experience [41]. They have the advantage of experiencing and gaining knowledge of health ICT sooner and therefore develop the capability to locate and recognize the value of new information, absorb it, and effectively apply it. These advantages become more salient under accelerated digitization, which introduces a host of new challenges to patients under difficult circumstances. For example, patients are less likely to receive face-to-face help from providers and their social circle. Consequently, underprivileged racial minority group patients who lack prior use are likely to have relatively low self-efficacy and fall further behind. In the context of the COVID-19 pandemic, users’ adoption of health technologies such as contact-tracing apps is influenced by their belief in their competence to use the app [42]. As a result, the racial digital divide may widen under accelerated digitization if underprivileged racial minority group patients do not feel confident in their ability to use health care portals and related technologies.

Second, it is also possible that the accelerated digitization results in narrowing of the racial digital divide when environmental factors take a dominant role. Many believe that the COVID-19 pandemic is a catalyst for digital transformation, especially in areas that traditionally had barriers to digital transformation such as health care [43]. This acceleration of digitization means that not only organizations are raising their IT budget but also individuals are much more receptive to technologies. In health care, the pandemic has stimulated innovation in terms of how care is delivered and how patients receive it. Physicians and patients increasingly use telehealth technologies such as contactless care, touchless health care, and telemedicine [44].

Despite the lack of prior use experience, underprivileged racial minority group patients may be more motivated to try health care portals during the pandemic, as alternative options were limited. This motivation aligns with Adaptation Level Theory, which posits that individuals perceive new stimuli or experiences as deviations from existing cognitions and revise their prior beliefs iteratively [45]. In our context, the pandemic served as a disruptive event, prompting underprivileged racial minority group patients to reevaluate their prior cognitions. Over time, as users engage repeatedly with the system, their cognitions stabilize, leading to sustained engagement and adaptation to new technology [46]. Pingree et al [47] suggested that when provided with equal opportunity and appropriate tools, racial minority group individuals will be as likely to adopt computer-based health applications. Hence, accelerated digitization provides a unique environment for underprivileged racial minority group patients to explore a greater quantity and variety of health ICT than before, which narrows the racial digital divide.

Behavioral factors such as mobile internet use may also play a role in affecting how patients use portals. The Pew Research Center [48] reported that underprivileged racial minority groups (87%) are more likely to own a smartphone (80%) and use mobile technologies such as data functions compared with White smartphone owners. According to Castells et al [49], underprivileged racial minority groups who were previously on the disadvantaged end of the digital divide have a higher adoption rate of mobile technology. Moreover, as minority groups enjoy equal access to smartphones, underprivileged racial minority group patients are more likely to engage in exploratory learning of unfamiliar health information using their preferred mobile devices [26]. As a result, they could use mobile channels to benefit more from web-based health care resources, such as patient portals, during accelerated digitization.

Because SCT provides conflicting predictions from both sides, there is a need for timely empirical evidence regarding the impact of accelerated digitization on the racial digital divide in health care.

## Methods

### Overview

Our patient portal use data were retrieved from a large AMC that offers a wide range of services and subspecialty care. The AMC is located in an urban area in the United States. Similar to the rest of the world, the city was severely disrupted by the COVID-19 pandemic. On March 11, 2020, the governor declared a state of emergency in response to the pandemic. The AMC took measures to limit the spread of SARS-CoV-2, including increasing personal protective equipment stockpiles, limiting visitors to both inpatient and outpatient settings, and halting nonemergency surgeries. In line with a broader trend, the AMC noticed a sharp decline in patients seeking in-person health care during the pandemic [50].

The AMC offers a patient portal as a central digital health gateway for its patients, which has been in place since 2012. The AMC’s patient portal is a secure web-based portal developed by one of the largest health ICT vendors and provides patients with a wide variety of tools. Patients can access their medical records, manage appointments, request prescription refills, view test results, pay bills, and communicate with their providers. The use data set contains each action performed by a user on the portal (eg, appointment details) and the functionality (eg, visit-related and bill-related actions), time stamp, and session information of the action. A session is a sequence of user actions that are coherent and uninterrupted so that the connections are kept alive by the server [51]. For each session, the data track the start and end time of a session and access via a mobile or web (desktop) device.

**Figure 1:**
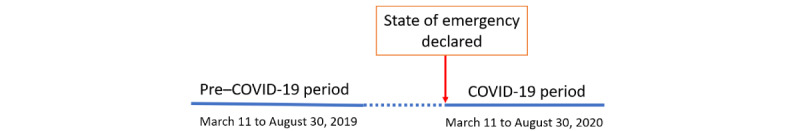
Period of sample data collection.

To quantify the impact of accelerated digitization on the racial digital divide, our identification strategy ensured that underprivileged racial minority group patients and White patients were affected by the pandemic to a similar extent after controlling for factors including age, income, location, health, COVID-19–related diagnosis, office visits, education attainment, and medical problems. In addition, we focused on the second-order effect use rather than the first-order effect access by limiting our sample to patients who had used the portal before the pandemic. We obtained all patients’ portal use data and their EHR data before August 30, 2020 (the end date of the data set). We limited the sample period of our study to 2 periods in 2019 and 2020 (Figure 1). The *COVID-19 period* was between March 11, 2020 (when the state of emergency was declared), and August 30, 2020. The corresponding *pre–COVID-19 period* was from March 11, 2019, to August 30, 2019. Among the 34,240 patients who used the patient portal during either of the sample periods (Figure 1), we excluded 5540 (16.18%) patients who did not use the patient portal at least once before March 11, 2020. We focused on the use behavior of 28,700 (83.82%) out of 34,240 existing patient portal users when COVID-19 started without including new patient portal users; all patients in our sample had logged in to the system at least once before the COVID-19 pandemic. Because the patient portal was integrated with the AMC’s EHR system, we merged the use data with the EHRs. The EHR data set included patient demographics, office visits, health vitals, diagnosis, insurance type, and medical problems. The merged sample included 26,742 patients with no missing data. Finally, we excluded patients who were identified as Asian, Native American, multiracial, and unknown ethnicities because they either represent only a small percentage of the patients or are not traditionally considered as underprivileged racial minority groups in the digital divide context [11,30,41,52-54]. Our final sample consisted of 25,612 patients belonging to 1 of the 3 racial groups: *Black or African American*, *Hispanic*, and *White patients*. Among the 25,612 patients, 5157 (20.14%) were Black or African American, 253 (0.99%) were Hispanic, and 20,202 (78.88%) were White. The proportion of underprivileged racial minority group patients in the sample was consistent with the overall underprivileged racial minority group population in the urban area.

We analyzed patient portal use at the monthly level. The main dependent variable was *PortalUsage_it_*, which is the number of portal use sessions in month *t* by patient *i.* We further divided the use sessions by the device channel; *Web_it_* is the number of sessions on the web (desktop) devices by patient *i* in month *t*, and *Mobile_it_* is the number of sessions on mobile (smartphone or tablet) devices by patient *i* in month *t*. Our Gini coefficient of function categories measured the variety of functions, and we categorized all patient actions into 1 of the 7 functional categories [51]: access, billing, messaging, record, telemedicine, visits, and others. For each patient and month, we computed the percentage of a user’s actions in each category. The Gini coefficient measures whether the use concentrates on only a few functionalities of the portal (high Gini) or on a wider variety of functionalities (low Gini).

Our main independent variables were *COVID_Period_it_*, *Minority_i_*, and their interaction *COVID_Period_it_*×*Minority_i_*. *COVID_Period_it_* indicator takes 1 if the month is during the pandemic period (ie, March 2020 to August 2020) and 0 if otherwise (ie, March 2019 to August 2019). *Minority_i_* indicator takes 1 if patient *i* is Black or Hispanic and 0 if White. The interaction of the 2 indicator variables indicates whether minority group patient portal use behaviors have changed relative to White patients during the pandemic period. In other words, the coefficient of the interaction term measures whether the racial digital divide widens or narrows during the pandemic.

We included control variables that can influence patients’ portal use. The first set of controls was related to their health conditions. Because minority groups are disproportionately affected by the pandemic, we controlled for patients’ COVID-19–related diagnoses or tests. The COVID-19 problem indicator takes 1 if a patient had a COVID-19–related International Classification of Diseases, 10th Revision (ICD-10), code in 2020, including a positive COVID-19 test result (ICD-10 code U07.1), exposure to SARS-CoV-2 (ICD-10 code Z20.828), or screening for COVID-19 (ICD-10 code Z11.59). The ICD-10 code Z20.828 is defined as follows: “Contact with and (suspected) exposure to other viral communicable diseases.” The American Academy of Family Physicians (AAFP) advises providers to assign this code when they believe a patient has been exposed to the novel coronavirus, but they are uncertain about whether to diagnose COVID-19 (eg, test results are not available) [55]. We controlled for patients’ overall health condition using their most recent measure of BMI and blood pressure (BP). We used the BP indicator to denote if the BP was above 120/80 mm Hg. The BMI and BP were winsorized at 1% and 99%, respectively, to account for outliers that are most likely due to recording errors (eg, using a centimeter instead of an inch as the unit of measurement for height). Because patient portal use may be associated with office visits [3,56], we also controlled for the number of outpatient office visits made by the patient in the previous month.

We also controlled for patients’ demographics, including their age, gender, marital status, and insurance. Patient marital status is a set of binary variables that includes married, significant other, divorced or separated, single, and others. Patient insurance status is a set of binary variables that indicates commercial, Medicaid, Medicare, self-pay, and others (eg, workers’ compensation). Finally, we found the average adjusted gross income of each patient’s zip code using the data from the Internal Revenue Service. We used the zip code–adjusted gross income, education attainment, and insurance status as proxies for the patient’s economic status. Prior research has consistently controlled for demographic covariates when studying eHealth use, and some studies found that demographic variables are associated with both well-being during the COVID-19 pandemic and access to and use of digital technologies [57-59].

### Statistical Analysis

We estimated panel data regression (equation 1) to investigate whether the racial divide of portal use widened during the COVID-19 pandemic:

*Ln(PortalUsage_it_ + 1)* = *β_1_Minority_i_* × *COVID_Period_t_* + *β_2_Minority_i_* + *β_3_COVID_Period_t_* + *ΓControls_it_* + *λOfficeVisit_it-1_* + *Month_t_* + *α_i_* + *ε_it_*
**(1)**

Conceptually, the model is akin to a 2-way repeated-measures ANOVA, where each patient is observed each month, and the main interest is the direction of the interaction between the 2 main factors (*COVID_Period* and *Minority*). As previously defined, the dependent variable, *PortalUsage_it_*, denotes the monthly portal use for each patient *i*. We took the natural logarithm of the session counts to mitigate skewness [3]. We later presented the results from count regression models as a robustness check. Each patient had 14 measurements of monthly portal use. The first 6 measurements occur before the pandemic, that is, the number of portal sessions from March 2019 to August 2019. During this period, the variable *COVID_Period* takes the value of 0. The last 6 measurements occur during the pandemic, that is, the number of portal sessions from March 2020 to August 2020. During this period, the variable *COVID_Period* takes the value 1. The β_1_ coefficient captures how the racial digital divide changed during the pandemic. *Controls_it_* include time-invariant patient controls of demographics and overall health conditions. *OfficeVisit_it_*_−_*_1_* is the number of office visits made in the previous month by a patient *i.* Lag avoids the simultaneity bias. We included monthly fixed effects (FEs) to account for seasonality.

We estimated 3 different models using the panel data set: pooled ordinary least squares (OLS) regression, random effect (RE), and FE. In the pooled OLS model, the individual effect is absorbed in the error term. In RE and FE models, the individual effect represents the unobserved, time-invariant patient-level heterogeneity in portal use. The RE model allows for the estimation of the effect of time-invariant regressors such as *Minority,* which is more efficient than the FE model, if the unobserved patient-specific effects are uncorrelated with other independent variables. However, the RE estimates are inconsistent if the above assumption does not hold. The FE model controls for all unobserved, patient-specific, and time-invariant heterogeneities; however, it cannot estimate the effects of time-invariant regressors, such as *Minority* and demographic controls.

### Ethics Approval

This study has been approved by the University of Cincinnati Institutional Review Board (2020-0723).

## Results

### Descriptive Statistics

Summary statistics and the definitions of the variables are shown in Table 1, and a correlation matrix is provided in Table 2.

**Table 1:** Variable definition and summary statistics.

Variable	Mean (SD)	Range	Definition
Portal usage	5.094 (11.369)	0-476	The number of patient portal sessions each month
Web usage	3.610 (8.695)	0-476	The number of patient portal sessions each month from web (desktop) devices
Mobile usage	1.484 (6.945)	0-352	The number of patient portal sessions each month from mobile devices
COVID_Period	0.509 (0.500)	0-1	Whether the month is March 2020 to August 2020
Minority	0.212 (0.409)	0-1	Whether the patient is Black or Hispanic
Age	53.028 (16.447)	15-102	The age of the patient in 2020
Gender (male=1)	0.377 (0.485)	0-1	The gender of the patient
BMI	30.763 (7.935)	12.87-71.01	The most recent BMI of the patient
Abnormal BP^a^	0.641 (0.480)	0-1	Whether the patient’s most recent BP is above 120/80
COVID problem	0.005 (0.069)	0-1	Whether the patient had a COVID-19–related ICD-10^b^ code
Office visits	0.547 (1.034)	0-25	The number of office visits made by the patient in the previous month
Income (US $1000)	76.07 (44.282)	18.635-931.273	Average adjusted gross income of the patient’s zip code from the IRS^c^
Education attainment	0.360 (0.161)	0-0.867	The percentage of the population (aged ≥25 years) with higher education degrees of the patient’s zip code from the USCB^d^
Medical problems	14.900 (12.219)	0-136	The number of active problems and conditions each month

^a^BP: blood pressure.

^b^ICD-10: International Classification of Diseases, 10th Revision.

^c^IRS: Internal Revenue Service.

^d^USCB: US Census Bureau.

**Table 2:** Correlation table.

		Portal usage	Web usage	Mobile usage	COVID_Period	Minority	Age	Gender	BMI	Abnormal BP^a^	COVID problem	Office visits	Income	Education attainment	Medical problems
**Portal usage**															
	*r*	1	0.79^b^	0.65	0.07	−0.04	0	0	0.03	0	0.02	0.27	0	0	0.13
	*P* value	–^c^	<.001	<.001	<.001	<.001	.53	.03	<.001	.08	<.001	<.001	.48	.51	<.001
**Web usage**															
	*r*	0.79	1	0.04	0.04	−0.05	0.06	0	0	0	0.01	0.21	0.02	0.02	0.11
	*P* value	<.001	–	<.001	<.001	<.001	<.001	.10	.43	.30	<.001	<.001	<.001	<.001	<.001
**Mobile usage**															
	*r*	0.65	0.04	1	0.07	0	−0.08	−0.01	0.04	−0.01	0.02	0.17	−0.02	0.02	0.06
	*P* value	<.001	<.001	–	<.001	.09	<.001	<.001	<.001	<.001	<.001	<.001	<.001	<.001	<.001
**COVID_Period**															
	*r*	0.07	0.04	0.07	1	0	−0.01	0	0	0	0	-0.04	0	0	0.03
	*P* value	<.001	<.001	<.001	–	.17	<.001	.62	.41	.44	.75	<.001	.89	.32	<.001
**Minority**															
	*r*	−0.04	−0.05	0	0	1	−0.19	−0.11	0.15	0.05	0.03	0.02	−0.2	−0.14	0.06
	*P* value	<.001	<.001	.09	.17	–	<.001	<.001	<.001	<.001	<.001	<.001	<.001	<.001	<.001
**Age**															
	*r*	0	0.06	−0.08	−0.01	−0.19	1	0.17	−0.11	0.13	0	0.07	0.12	0.12	0.24
	*P* value	.53	<.001	<.001	<.001	<.001	–	<.001	<.001	<.001	.02	<.001	<.001	<.001	<.001
**Gender**															
	*r*	0	0	−0.01	0	−0.11	0.17	1	−0.11	0.08	−0.02	0.01	0.04	0.04	−0.01
	*P* value	.03	.10	<.001	.62	<.001	<.001	–	<.001	<.001	<.001	<.001	<.001	<.001	.002
**BMI**															
	*r*	0.03	0	0.04	0	0.15	−0.11	−0.11	1	0.17	0.01	0.03	−0.13	−0.13	0.1
	*P* value	<.001	.43	<.001	.41	<.001	<.001	<.001	–	<.001	<.001	<.001	<.001	<.001	<.001
**Abnormal BP**															
	*r*	0	0	−0.01	0	0.05	0.13	0.08	0.17	1	0	0.01	−0.03	−0.03	0.04
	*P* value	.08	.30	<.001	.44	<.001	<.001	<.001	<.001	–	.20	<.001	<.001	<.001	<.001
**COVID problem**															
	*r*	0.02	0.01	0.02	0	0.03	0	−0.02	0.01	0	1	0.02	0	−0.01	0.02
	*P* value	<.001	<.001	<.001	.75	<.001	.02	<.001	<.001	.20	–	<.001	.03	.003	<.001
**Office visits**															
	*r*	0.27	0.21	0.17	−0.04	0.02	0.07	0.01	0.03	0.01	0.02	1	−0.01	0	0.23
	*P* value	<.001	<.001	<.001	<.001	<.001	<.001	<.001	<.001	<.001	<.001	–	<.001	.54	<.001
**Income**															
	*r*	0	0.02	−0.02	0	−0.2	0.12	0.04	−0.13	−0.03	0	−0.01	1	0.71	−0.04
	*P* value	.48	<.001	<.001	.89	<.001	<.001	<.001	<.001	<.001	.03	<.001	–	<.001	<.001
**Education attainment**															
	*r*	0	0.02	.02	0	−0.14	0.12	0.04	−0.13	−0.03	−0.01	0	0.71	1	−0.03
	*P* value	.51	<.001	<.001	.32	<.001	<.001	<.001	<.001	<.001	.003	.54	<.001	–	<.001
**Medical problems**															
	*r*	0.13	0.11	0.06	0.03	0.06	0.24	−0.01	0.10	0.04	0.02	0.23	−0.04	−0.03	1
	*P* value	<.001	<.001	<.001	<.001	<.001	<.001	.002	<.001	<.001	<.001	<.001	<.001	<.001	–

^a^BP: blood pressure.

^b^The correlation is significant at a level of .01 (two-tailed).

^c^Not applicable.

### Data Analysis

Despite the strengths and weaknesses of each model, we obtained consistent results from all 3 models reported for comparison in Table 3.

**Table 3:** Effect of the COVID-19 pandemic on the digital divide^a^.

DV^b^: ln(Usage)			OLS^c^	RE^d^		FE^e^
**COVID_Period**						
	β (SE)	.228 (.004)		.194 (.004)	.156 (.004)	
	*P* value	<.001		<.001	<.001	
**Minority**						
	β (SE)	−.158 (.007)		−.168 (.011)	N/A^f^	
	*P* value	<.001		<.001	N/A	
**COVID_Period×Minority**						
	β (SE)	.028 (.009)		.037 (.007)	.043 (0.007)	
	*P* value	.002		<.001	<.001	
**Age**						
	β (SE)	−.004 (.0002)		−.004 (.0003)	N/A	
	*P* value	<.001		<.001	N/A	
**Male**						
	β (SE)	−.046 (.004)		−.041 (.009)	N/A	
	*P* value	<.001		<.001	N/A	
**BMI**						
	β (SE)	.004 (.0003)		.004 (.001)	N/A	
	*P* value	<.001		<.001	N/A	
**Abnormal BP^g^**						
	β (SE)	−.004 (.004)		−.006 (.009)	N/A	
	*P* value	.32		.52	N/A	
**COVID problem**						
	β (SE)	.197 (.033)		.224 (.070)	N/A	
	*P* value	<.001		.002	N/A	
**Office visit**						
	β (SE)	.317 (.003)		.184 (.002)	.145 (.002)	
	*P* value	<.001		<.001	<.001	
**Income**						
	β (SE)	−.0003 (.0001)		−.0002 (.0001)	N/A	
	*P* value	<.001		.06	N/A	
**Education attainment**						
	β (SE)	.103 (.017)		.121 (.036)	N/A	
	*P* value	<.001		.001	N/A	
**Medical problems**						
	β (SE)	.011 (.0002)		.016 (.0003)	.042 (.001)	
	*P* value	<.001		<.001	<.001	
**Constant**						
	β (SE)	.453 (.015)		.503 (.030)	N/A	
	*P* value	<.001		<.001	N/A	
Marital status FE			Yes	Yes		Yes
Insurance FE			Yes	Yes		Yes
Month FE			Yes	Yes		Yes
Observations			301,281	301,281		301,281
*R* ^2^			0.13	0.066		0.052

^a^Using a panel data set, we estimated pooled OLS, RE, and FE models to examine the relationship between the COVID_Period×Minority (main independent variable) and patient portal use (DV). Robust SEs are reported in parentheses.

^b^DV: dependent variable.

^c^OLS: ordinary least squares.

^d^RE: random effect.

^e^FE: fixed effect.

^f^N/A: not applicable.

^g^BP: blood pressure.

First, we examined the effects of the 2 main factors, *COVID_Period* and *Minority*. Our results show a significant increase in patient portal use during the pandemic. The coefficient of *COVID_Period* is positive and significant across the 3 models (OLS, β=.228; *P*<.001; RE, β=.194; *P*<.001; FE, β=.156; *P*<.001). The results indicate that monthly session use increased by 16% to 23% after the onset of the pandemic. The coefficient of *Minority* is negative and significant (OLS, β=−.158; *P*<.001; RE, β=−.168; *P*<.001), indicating that there was a pre–COVID-19 racial disparity in portal use that disadvantaged underprivileged racial minority group patients. Specifically, the RE model indicates that underprivileged racial minority group patients’ monthly portal use was 16.8% lower than that of White patients.

We evaluated whether the racial digital divide was widening owing to the widespread digital acceleration triggered by the pandemic. The interaction of *COVID_Period* is positive and significant across the 3 models (OLS, β=.028; *P*=.002; RE, β=.037; *P*<.001; FE, β=.043; *P*<.001). The results suggest that the racial disparity of patient portal use did not widen during the COVID-19 pandemic; instead, the pandemic reduced the gap by 22% (.037/.168) as shown by the RE model.

The regression results revealed a few other important patterns regarding patient portal use. We found that female and younger users were more likely to use the patient portal. Patients with worse health conditions, as indicated by higher BMI, COVID-19–related diagnosis, and recent office visits, were also more frequent users. Finally, there was no effect of income on patient portal use, at least in the direction that most would expect; patients living in more affluent neighborhoods were no more likely to use the patient portal.

We also examined the patient portal use from distinct types of internet access (mobile vs web). The model specification remains the same with 1 change—the dependent variable is the number of sessions viewed from a mobile interface or a web (desktop) interface. Table 4 shows the results. In the RE model, the coefficient of *COVID_Period×Minority* is −.013 (*P=*.06) for the web channel and .042 (*P<*.001) for the mobile channel. The racial digital divide is *increasing* for the web channel and *decreasing* for the mobile channel. The same pattern can be observed for the other 2 specifications. Table 4 provides convincing evidence that the closing of racial disparity is largely driven by those who use their portals through the mobile channel.

To examine meaningful use, we studied whether COVID-19–induced digital acceleration has a similar effect on the variety of the portal functionalities used, as indicated by the Gini coefficient. Table 5 shows the results from the regression model (equation 2), which uses the Gini coefficient of the portal action categories as the dependent variable:

*Gini_it_* = *β_1_Minority_i_* × *COVID_Period_t_* + *β_2_Minority_i_* + *β_3_COVID_Period_t_* + *ΓControls_it_* + *λOfficeVisit_it-1_* + *Month_t_* + *α_i_* + *ε_it_*
**(2)**

**Table 4:** Change of digital divide: internet access mode^a^.

DV^b^: ln(Usage)		OLS^c^		RE^d^		FE^e^	
		Web	Mobile	Web	Mobile	Web	Mobile
**COVID_Period**							
	β (SE)	.124 (.004)	.176 (.003)	.100 (.003)	.157 (.002)	.075 (.003)	.142 (.002)
	*P* value	<.001	<.001	<.001	<.001	<.001	<.001
**Minority**							
	β (SE)	−.111 (.006)	−.066 (.004)	−.120 (.010)	−.075 (.009)	N/A^f^	N/A
	*P* value	<.001	<.001	<.001	<.001	N/A	N/A
**COVID_Period×Minority**							
	β (SE)	−.020 (.008)	.037 (.007)	−.013 (.007)	.042 (.005)	−.008 (.006)	.044 (.004)
	*P* value	.02	<.001	.06	<.001	.19	<0.001
**Age**							
	β (SE)	.003 (.0001)	−.007 (.0001)	.002 (.0003)	−.008 (.0003)	N/A	N/A
	*P* value	<.001	<.001	<.001	<.001	N/A	N/A
**Male**							
	β (SE)	−.054 (.004)	.010 (.003)	−.052 (.008)	.015 (.007)	N/A	N/A
	*P* value	<.001	<.001	<.001	.04	N/A	N/A
**BMI**							
	β (SE)	.0004 (.0002)	.004 (.0002)	.0001 (.001)	.003 (.0005)	N/A	N/A
	*P* value	.07	<.001	.85	<.001	N/A	N/A
**Abnormal BP^g^**							
	β (SE)	.001 (.004)	−.004 (.003)	−.002 (.009)	−.001 (.008)	N/A	N/A
	*P* value	.88	.18	.83	.84	N/A	N/A
**COVID problem**							
	β (SE)	.062 (.029)	.195 (.028)	.077 (.064)	.211 (.073)	N/A	N/A
	*P* value	.04	<.001	.23	.004	N/A	N/A
**Office visit**							
	β (SE)	.224 (.003)	.133 (.002)	.129 (.002)	.066 (.002)	.105 (.002)	.056 (.001)
	*P* value	<.001	<.001	<.001	<.001	<.001	<.001
**Income**							
	β (SE)	−.0001 (.0001)	−.0003 (.00004)	−.0001 (.0001)	−.0002 (.0001)	N/A	N/A
	*P* value	.25	<.001	.69	.01	N/A	N/A
**Education attainment**							
	β (SE)	.109 (.016)	.008 (.011)	.121 (.035)	.020 (.029)	N/A	N/A
	*P* value	<.001	.45	.001	.49	N/A	N/A
**Medical problems**							
	β (SE)	.008 (.0002)	.004 (.0001)	.012 (.0003)	.009 (.0003)	.029 (.001)	.020 (.001)
	*P* value	<.001	<.001	<.001	<.001	<.001	<.001
**Constant**							
	β (SE)	.247 (.014)	.213 (.011)	.279 (.029)	.236 (.026)	N/A	N/A
	*P* value	<.001	<.001	<.001	<.001	N/A	N/A
Marital Status FE		Yes	Yes	Yes	N/A	N/A	N/A
Insurance FE		Yes	Yes	Yes	N/A	N/A	N/A
Month FE		Yes	Yes	Yes	N/A	N/A	N/A
Observations		301,281	301,281	301,281	301,281	301,281	301,281
R^2^		0.087	0.077	0.037	0.052	0.026	0.051

^a^Using a panel data set, we estimate pooled OLS, RE, and FE models to examine whether the relationship between the COVID_Period×Minority (main independent variable) and patient portal use (DV) differs by internet access mode. Robust SEs are reported in parentheses.

^b^DV: dependent variable.

^c^OLS: ordinary least squares.

^d^RE: random effect.

^e^FE: fixed effect.

^f^N/A: not applicable.

^g^BP: blood pressure.

**Table 5:** Effect of COVID-19 on digital divide: variety of functionality^a^.

DV^b^: Gini (Functionality)			OLS^c^		RE^d^		FE^e^
**COVID_Period**							
	β (SE)	−.002 (.001)		−.001 (.0005)		−.0004 (.0005)	
	*P* value	<.001		.01		.44	
**Minority**							
	β (SE)	.002 (0.001)		.002 (0.001)		N/A^f^	
	*P* value	.002		.05		N/A	
**COVID_Period×Minority**							
	β (SE)	−.004 (.001)		−.004 (.001)		−.003 (.001)	
	*P* value	<.001		<.001		.001	
**Age**							
	β (SE)	−.0003 (.00002)		−.0004 (.00003)		N/A	
	*P* value	<.001		<.001		N/A	
**Male**							
	β (SE)	.004 (.0005)		.004 (.001)		N/A	
	*P* value	<.001		<.001		N/A	
**BMI**							
	β (SE)	−.00001 (.00003)		.00003 (.00005)		N/A	
	*P* value	.71		.58		N/A	
**Abnormal BP^g^**							
	β (SE)	.0004 (.0005)		.0004 (.001)		N/A	
	*P* value	.38		.65		N/A	
**COVID problem**							
	β (SE)	.021 (.003)		.023 (.006)		N/A	
	*P* value	<.001		<.001		N/A	
**Ln(Usage+1)**							
	β (SE)	−.128 (.0003)		−.127 (.0003)		−.126 (.0003)	
	*P* value	<.001		<.001		.001	
**Office visit**							
	β (SE)	.004 (.0003)		.003 (.0003)		.001 (.0003)	
	*P* value	<.001		<.001		.001	
**Mobile usage %**							
	β (SE)	.008 (.001)		−.015 (.001)		−.042 (.001)	
	*P* value	<.001		<.001		.001	
**Income**							
	β (SE)	.00002 (.00001)		.00002 (.00001)		N/A	
	*P* value	.007		.15		N/A	
**Education attainment**							
	β (SE)	−.018 (.002)		−.018 (.003)		N/A	
	*P* value	<.001		<.001		N/A	
**Medical problems**							
	β (SE)	.0003 (.00002)		.0003 (.00003)		.0004 (.0001)	
	*P* value	<.001		<.001		.001	
**Constant**							
	β (SE)	.928 (.002)		.931 (.003)		N/A	
	*P* value	<.001		<.001		N/A	
Marital status FE			Yes		Yes		Yes
Insurance FE			Yes		Yes		Yes
Month FE			Yes		Yes		Yes
Observations			301,281		301,281		301,281
*R* ^2^			0.575		0.527		0.495

^a^Using a panel data set, we estimated pooled OLS, RE, and FE models to examine whether the relationship between COVID_Period×Minority (main independent variable) and variety of functionality (DV) differs by internet access mode. Robust SEs are reported in parentheses.

^b^DV: dependent variable.

^c^OLS: ordinary least squares.

^d^RE: random effect.

^e^FE: fixed effect.

^f^N/A: not applicable.

^g^BP: blood pressure.

The coefficients of *COVID_Period* are negative and significant for the OLS and RE specifications, suggesting that patients were more likely to use a wider range of functionalities available in the patient portal during the pandemic. Table 5 also shows that underprivileged racial minority group patients have a higher Gini, meaning that they tend to concentrate their use on a narrower set of functionalities. Importantly, the coefficients of *COVID_Period×Minority* are negative and significant at *P*<.001 or *P*=.001 level for 3 specifications, indicating that the pandemic-induced digital acceleration is also closing the racial digital gap, that is, underprivileged racial minority group patients were increasingly using a wider variety of functionalities and more so than their White counterpart.

We conducted a series of robustness checks that included a falsification test that showed that closing the gap is not part of a long-term trend, an alternative model specification using Poisson regression, and a propensity score–matching analysis to further mitigate endogeneity concerns. Multimedia Appendix 1 [11,30,41,52-54,60-64] provides further details.

## Discussion

### Principal Findings and Implications

Our study provides timely empirical evidence that accelerated digitization has not hardened the divide as many feared; instead, it may have provided a push for the use of telehealth among underprivileged racial minority groups. The effect is mostly driven by use through mobile devices and cannot be explained by the pre–COVID-19 trends. Moreover, the use of a variety of functionalities on the patient portal by underprivileged racial minority groups also expanded faster than that by White patients. Overall, these findings provide new theoretical insights from an SCT perspective. We show that mobile internet use patterns, combined with accelerated digitization, which represents a sufficiently large shock to the environment, outweigh personal factors such as self-efficacy in driving behavioral changes on patient portal use and thus in combating the digital divide.

Our findings have major implications for managing the digital divide and technology use in health care. First, there are reasons for optimism although the COVID-19 pandemic has been cited as a “great magnifier” of preexisting racial inequality in health [65]. Since the start of the pandemic-induced accelerated digitization, underprivileged racial minority group patients who underutilized telehealth have embraced it at a faster pace than White patients. This means that telehealth has the potential to become a “great equalizer” for reducing digital racial health inequality. As such, across-the-board incentives to facilitate the use of patient portals can be an effective measure to address health inequality, especially during severe disruptions to in-person health care.

Second, in the United States, much effort in combating the digital divide has focused on the broadband connectivity gap. For example, the former Federal Communications Commission Commissioner Mignon Clyburn called broadband access a “super-determinant” of health during the launch of the *Mapping Broadband Health in America Platform* [66], yet the platform only considers fixed residential connections. The transformative potential of mHealth is overlooked [67]. Our study adds to the public policy debate on mobile versus fixed connections [68]—at least in the telehealth setting, policy makers could prioritize closing the mobile or wireless internet gap. Moreover, health care providers can make web-based health resources and similar features accessible through mobile interfaces and add training programs so that patients can install and benefit from mHealth apps. These efforts would contribute to the closing of the racial digital divide and solidify the gains in patient portal use.

Third, we make a conservative claim that greater use of patient portals is associated with increased exposure to health knowledge. Our results suggest that underprivileged racial minority groups lag in health knowledge; however, accelerated digitization has facilitated greater use and thereby enabled opportunities for learning to accrue. Digital divide research in health care has made it clear that the lack of access to portals has disadvantaged underprivileged racial minority groups and has made it challenging for them to gain health literacy at the same rate as others [69]. Despite this, our results indicate that as long as they have access, underprivileged racial minority groups are using the portal and *catching up*—they used the portal more frequently and expanded their use to a wider range of functionalities during the post–COVID-19 period. These information-seeking and exploratory behaviors are key indicators of learning [70,71] and central to the development of personal health literacy, whose goal is to use health information to make well-informed decisions [72]. Therefore, the shrinkage of the digital divide gap found in our study highlights an opportunity to conduct *knowledge gap* research, including across racial lines, for improving personal health literacy that is known to improve health care quality.

### Limitations

This study is not without limitations. First, we were unable to collect patients’ treatment outcome information. We could not observe whether more frequent and meaningful use of the web-based portal improved patients’ long-term health conditions in our sample; however, Bao et al [3] found that portal use has positive causal effects on patient health outcomes. Second, our analysis is limited to a single AMC providing limited services to the rural population. Although this hospital offers a large and diverse patient sample, additional research is needed to determine whether this gap shrinks at a broader level. However, we have controlled for various demographic variables in our analysis, which helped mitigate the potential biases arising from the limited representativeness of our sample. Third, this study used a pretest-posttest design, which rests on the assumption that patients’ prepandemic use provides a valid counterfactual assumption. As we provide support for this identifying assumption, and we are not aware of other unrelated shocks that may affect patient portal use, future field experiments may be carried out to complement our findings.

### Conclusions

The COVID-19–induced digitization and its consequences are likely to persist beyond the pandemic. Mixed views exist as to what an abrupt digital shift entails; some are concerned that it may expose and aggravate a persistent digital divide, whereas others see it as a silver lining [73]. In this study, we use the COVID-19 pandemic as a natural experiment to investigate whether such accelerated digitization has widened the racial digital divide in telehealth. We used a longitudinal patient portal use data set from a large urban AMC to explore the effect of accelerated digitization on the racial digital gap in health care. We found that Black and Hispanic patients had lower patient portal use compared with White patients before the pandemic; however, contrary to widespread belief, the gaps in both quantity and variety of use are shrinking following the outbreak of the pandemic. Interestingly, most of the increased use is driven by the mobile channel. These findings provide new insights into the digital behaviors among underprivileged racial minority groups during accelerated digitization. They also offer policy makers an opportunity to identify new strategies to help close the racial digital gap in the postpandemic world and call for *knowledge gap* research for improving personal health literacy that is known to improve health care quality.

## Appendix

Multimedia Appendix 1 Robustness checks.

## Abbreviations

AAFP: American Academy of Family Physicians
AMC: academic medical center
BP: blood pressure
EHR: electronic health record
FE: fixed effect
ICD-10: International Classification of Diseases, 10th Revision
ICT: information and communications technologies
IS: information systems
mHealth: mobile health
OLS: ordinary least squares
RE: random effect
SCT: social cognitive theory
