# Supplementary material for: The Impact of Accelerated Digitization on Patient Portal Use by Underprivileged Racial Minority Groups During COVID-19: Longitudinal Study
Source: J Med Internet Res. 2023 Aug 9;25:e44981. doi: 10.2196/44981 (PMC10414031; doi:10.2196/44981)
Supplement: Multimedia Appendix 1 [file jmir_v25i1e44981_app1.docx]

## Multimedia Appendix 1. Robustness Checks

Our main findings are generated from a pre-post design; therefore, the underlying identifying assumption is that in the absence of COVID-induced digital acceleration, the digital divide would not have closed. A natural concern related to our research design is that the closing of the racial digital gap is not due to accelerated digitalization but rather part of a long-term trend. We conduct an in-time placebo test by repeating the main analysis using an intervention period that is earlier than the actual COVID period [60]. Specifically, we use the 3/11/2019 – 8/31/2019 as the “placebo” COVID treatment period and use 3/11/2018 – 8/31/2018 as the “control” period. Table A1 shows the results. The coefficients of the “placebo” COVID period are negative and significant across all specifications, which stand opposite to our main results. Stated differently, before COVID, the racial digital divide existed and was widening. Therefore, we can conclude that COVID’s eﬀect on closing the racial gap was not the result of a pre-treatment trend.

Table A1. Falsification Test

| **DV: ln (Usage)** | **OLS** | **Random Effect** | **Fixed Effect** |
| --- | --- | --- | --- |
| COVID_Period (Placebo) | 0.138^***^ | 0.139^***^ | 0.140^***^ |
|  | (0.004) | (0.004) | (0.003) |
| Minority | -0.091^***^ | -0.085^***^ |  |
|  | (0.007) | (0.012) |  |
| COVID_Period (Placebo) × Minority | -0.019^**^ | -0.020^**^ | -0.020^***^ |
|  | (0.010) | (0.008) | (0.007) |
| Age | -0.002^***^ | -0.002^***^ |  |
|  | (0.0002) | (0.0003) |  |
| Male | -0.045^***^ | -0.043^***^ |  |
|  | (0.004) | (0.009) |  |
| BMI | 0.006^***^ | 0.006^***^ |  |
|  | (0.0003) | (0.001) |  |
| Abnormal BP | 0.013^***^ | 0.011 |  |
|  | (0.004) | (0.009) |  |
| COVID problem | 0.146^***^ | 0.175^**^ |  |
|  | (0.035) | (0.073) |  |
| Office visits | 0.314^***^ | 0.197^***^ | 0.166^***^ |
|  | (0.003) | (0.002) | (0.002) |
| Income | -0.0001^**^ | -0.0001 |  |
|  | (0.00004) | (0.0001) |  |
| Constant | 0.483^***^ | 0.511^***^ |  |
|  | (0.015) | (0.030) |  |
|  | | | |
| Marital Status FE | Yes | Yes | -- |
| Insurance FE | Yes | Yes | -- |
| Month FE | Yes | Yes | Yes |
| Observations | 254,916 | 254,916 | 254,916 |
| R^2^ | 0.111 | 0.054 | 0.042 |

*Note:* Robust standard errors in parentheses are clustered at the patient level. ^*^*P*<.1; ^**^ *P* <.05; ^***^ *P* <.01

We analyze portal usage frequency by using a log-transformed dependent variable. As a robustness check, we use Poisson regression models by treating patient portal usage as a count variable. As Tables A2 and A3 show, we obtain equivalent results to the main analysis.

Table A2. Effect of COVID on Digital Divide (Poisson Model)

| **DV: Usage** | **OLS** | **Random Effect** | **Fixed Effect** |
| --- | --- | --- | --- |
| COVID_Period | 0.812*** | 0.790*** | 0.790*** |
|  | (0.004) | (0.004) | (0.004) |
| Minority | -0.266*** | -0.266*** |  |
|  | (0.003) | (0.018) |  |
| COVID_Period × Minority | 0.016*** | 0.032*** | 0.032*** |
|  | (0.004) | (0.004) | (0.004) |
| Age | -0.004*** | -0.004*** |  |
|  | (0.000) | (0.001) |  |
| Male | -0.025*** | -0.031* |  |
|  | (0.002) | (0.015) |  |
| BMI | 0.008*** | 0.009*** |  |
|  | (0.000) | (0.001) |  |
| Abnormal BP | -0.005** | -0.016 |  |
|  | (0.002) | (0.015) |  |
| COVID problem | 0.449*** | 0.541*** |  |
|  | (0.009) | (0.101) |  |
| Office visits | 0.272*** | 0.144*** | 0.140*** |
|  | (0.000) | (0.001) | (0.001) |
| Income | -0.000*** | -0.000 |  |
|  | (0.000) | (0.000) |  |
| Constant | 0.961*** | 0.974*** |  |
|  | (0.007) | (0.048) |  |
|  | | | |
| Marital Status FE | Yes | Yes | -- |
| Insurance FE | Yes | Yes | -- |
| Month FE | Yes | Yes | Yes |
| Observations | 310,500 | 310,500 | 310,500 |

*Note:* Robust standard errors in parentheses are clustered at the patient level. ^*^ *P* <.1; ^**^ *P* <.05; ^***^ *P* <.01

Table A3. Change of Digital Divide: Internet Access Mode (Poisson Model)

| **DV: Usage** | **Web**  **(OLS)** | **Mobile**  **(OLS)** | **Web**  **(Random Effect)** | **Mobile**  **(Random Effect)** | **Web**  **(Fixed Effect)** | **Mobile**  **(Fixed Effect)** |
| --- | --- | --- | --- | --- | --- | --- |
| COVID_Period | 0.617*** | 1.325*** | 0.604*** | 1.279*** | 0.605*** | 1.279*** |
|  | (0.005) | (0.009) | (0.005) | (0.009) | (0.005) | (0.009) |
| Minority | -0.256*** | -0.217*** | -0.256*** | -0.230*** |  |  |
|  | (0.004) | (0.007) | (0.020) | (0.059) |  |  |
| COVID_Period × Minority | -0.054*** | 0.039*** | -0.040*** | 0.068*** | -0.040*** | 0.068*** |
|  | (0.005) | (0.008) | (0.005) | (0.008) | (0.005) | (0.008) |
| Age | 0.005*** | -0.027*** | 0.006*** | -0.028*** |  |  |
|  | (0.000) | (0.000) | (0.001) | (0.002) |  |  |
| Male | -0.057*** | 0.072*** | -0.079*** | 0.096* |  |  |
|  | (0.002) | (0.003) | (0.016) | (0.048) |  |  |
| BMI | 0.004*** | 0.018*** | 0.005*** | 0.017*** |  |  |
|  | (0.000) | (0.000) | (0.001) | (0.003) |  |  |
| Abnormal BP | -0.007*** | -0.003 | -0.019 | -0.031 |  |  |
|  | (0.002) | (0.003) | (0.016) | (0.048) |  |  |
| COVID problem | 0.242*** | 0.804*** | 0.325** | 1.108*** |  |  |
|  | (0.012) | (0.014) | (0.111) | (0.330) |  |  |
| Office visits | 0.249*** | 0.321*** | 0.135*** | 0.150*** | 0.131*** | 0.149*** |
|  | (0.000) | (0.001) | (0.001) | (0.001) | (0.001) | (0.001) |
| Income | 0.000*** | -0.002*** | 0.000 | -0.002*** |  |  |
|  | (0.000) | (0.000) | (0.000) | (0.001) |  |  |
| Constant | 0.344*** | 0.379*** | 0.342*** | 0.436** |  |  |
|  | (0.008) | (0.013) | (0.052) | (0.157) |  |  |
|  | | | | | | |
| Marital Status FE | Yes | Yes | Yes | Yes | -- | -- |
| Insurance FE | Yes | Yes | Yes | Yes | -- | -- |
| Month FE | Yes | Yes | Yes | Yes | Yes | Yes |
| Observations | 310,500 | 310,500 | 310,500 | 310,500 | 299,220 | 99,132 |

*Note:* Robust standard errors in parentheses are clustered at the patient level. ^*^ *P* <.1; ^**^ *P* <.05; ^***^ *P* <.01

In addition, we perform a propensity score matching (PSM) analysis to further mitigate the endogeneity concerns. The treatment is equal to one if a patient is a URM and zero otherwise. Because our goal is to assess whether the two groups’ patient portal usage systematically differs during COVID, we aim to create a control group that has similar characteristics to the treatment group before COVID. Specifically, we employ a single nearest neighbor matching using the *psmatch2* model in Stata and match patients in the treatment group with patients in the control group on a range of factors, including current age, BMI, abnormal blood pressure, COVID diagnosis, number of office visits, ZIP-code level income, marital status, insurance status, and gender. For time-variant variables such as the number of office visits, we take the average value for each patient before COVID; all others are time-invariant factors in our sample. We adopt the Standardized Mean Difference (SMD) as the imbalance measure; an SMD smaller than 0.1 suggests that the matched dataset is well-balanced [61,62]. Table A4 shows that the average SMD dropped from 0.228 before matching to 0.066 after matching, with the SMD significantly reduced for all individual covariates, indicating that the covariates are balanced between the treatment and the control groups. Table A5 reports the main regression results of Eq. (1) using the matched sample. The results are qualitatively the same as the unmatched sample. In unreported results, the findings of Internet access mode and the variety of functionality are consistent when using the matched sample.

Table A4. Standardized Mean Difference Before and After Propensity Score Matching (PSM)

| **Variables** | **Before Matching** | **After Matching** |
| --- | --- | --- |
| Age | 0.453 | 0.127 |
| BMI | -0.345 | -0.083 |
| Abnormal BP | -0.118 | -0.033 |
| COVID_Period | -0.091 | -0.047 |
| Office visits | -0.046 | -0.019 |
| Income | 0.721 | 0.251 |
| Marital status | 0.664 | 0.151 |
| Insurance status | 0.558 | 0.150 |
| Gender | 0.257 | 0.097 |
| **Average** | **0.228** | **0.066** |

Our robustness check includes other unreported analyses to rule out alternative explanations. First, we check whether the effect of *COVID_Period* is driven by other acute conditions accompanying COVID but is not captured by the indicator *COVID_Problem*. We use the chronic condition indicator by AHRQ to classify the patients in the sample into two categories, those with chronic diseases and those with only acute diseases [63]. We repeat the analysis of monthly portal sessions using this split sample. The coefficients on *COVID_Period * Minority* are significant and positive only for the chronic patient groups. We conclude that the reduction of the racial gap cannot be attributed to acute conditions arising during COVID. Second, we rule out the possibility that inexperienced URM users took many different sessions to complete a single task. We use the log of total active session length (in seconds), rather than the number of sessions, as the dependent variable. The results are qualitatively the same.

While the main analysis only includes three races, it would be beneficial to perform robustness checks using the entire population to verify the main results. This is because, while Hispanic individuals only make up a small proportion (1%) of the data sample, their inclusion in the sample suggests that other races should also be present. It would be valuable to consider the full range of races represented in the data to ensure the results are as robust as possible. The following table shows the race distribution in our dataset. The biggest race that was excluded in our previous analysis is Asian (2.20%).

Table A5. Effect of COVID on Digital Divide (PSM)

| **DV: ln (Usage)** | **OLS** | **Random Effect** | **Fixed Effect** |
| --- | --- | --- | --- |
| COVID_Period | 0.330*** | 0.326*** | 0.322*** |
|  | (0.009) | (0.014) | (0.014) |
| Minority | -0.158*** | -0.161*** |  |
|  | (0.008) | (0.015) |  |
| COVID_Period × Minority | 0.040*** | 0.045** | 0.048** |
|  | (0.012) | (0.017) | (0.017) |
| Age | -0.001*** | -0.000 |  |
|  | (0.000) | (0.000) |  |
| Male | -0.056*** | -0.053*** |  |
|  | (0.006) | (0.014) |  |
| BMI | 0.007*** | 0.008*** |  |
|  | (0.000) | (0.001) |  |
| Abnormal BP | -0.036*** | -0.043** |  |
|  | (0.006) | (0.014) |  |
| COVID problem | 0.079* | 0.093 |  |
|  | (0.032) | (0.064) |  |
| Office visits | 0.309*** | 0.202*** | 0.173*** |
|  | (0.004) | (0.004) | (0.004) |
| Income | 0.005 | 0.007 |  |
|  | (0.008) | (0.016) |  |
| Constant | 0.284*** | 0.288*** | 0.422*** |
|  | (0.038) | (0.080) | (0.007) |
|  | | | |
| Marital Status FE | Yes | Yes | -- |
| Insurance FE | Yes | Yes | -- |
| Month FE | Yes | Yes | Yes |
| Observations | 134,008 | 134,008 | 134,008 |
| R^2^ | 0.134 | 0.128 | 0.111 |

*Note:* Robust standard errors in parentheses are clustered at the patient level. ^*^ *P* <.1; ^**^ *P* <.05; ^***^ *P* <.01

According to the U.S. Census data, Asian households have a higher median income compared to other racial and ethnic groups in the United States. This can result in greater access to resources, including healthcare. On the educational side, Asian Americans have higher levels of educational attainment compared to other racial and ethnic groups. This can also result in greater access to resources and opportunities. Moreover, Asian Americans often bring with them cultural and linguistic capital that can be advantageous in navigating the healthcare system. For example, they may have a greater understanding of alternative medicine or traditional healing practices, which can supplement their healthcare. For these reasons, in the healthcare literature, African Americans and Hispanics are usually considered underprivileged racial minorities (URMs). Therefore, we follow common practices in healthcare literature, such as [11, 30, 41, 52-54, 64], and treat African Americans and Hispanics as the URM.

Table A6. Race Distribution of the Patients in our Dataset

| **Race** | **Count** | **Percentage** |
| --- | --- | --- |
| American Indian or Alaska Native | 53 | 0.20% |
| Asian | 588 | 2.20% |
| Black or African American | 5157 | 19.28% |
| Hispanic | 253 | 0.95% |
| Multiracial | 167 | 0.62% |
| Native Hawaiian or Other Pacific Islander | 23 | 0.09% |
| Other | 255 | 0.95% |
| Patient Refused | 37 | 0.14% |
| Unknown | 7 | 0.03% |
| White or Caucasian | 20202 | 75.54% |
| Total | 26742 | 100.00% |

Nevertheless, it is important to recognize the diversity of experiences among Asian Americans and to address the unique healthcare needs and challenges of different Asian ethnic groups in healthcare policy and research. For this reason, we investigated the population distribution of the city where this hospital is located. The following table shows the poverty rate for different races in the city where the hospital is located. As the data shows, Asian’s poverty rate is about 3% higher than White. Islander and Native groups have very low poverty rates.

Table A7. Poverty Rate in the Local City^^[[1]](#footnote-1)^^

| **Name** | **Population** | **Poverty** | **Poverty Rate** |
| --- | --- | --- | --- |
| Black | 95467 | 40202 | 42.11% |
| White | 129304 | 21741 | 16.81% |
| Hispanic | 7815 | 4400 | 56.30% |
| Multiple | 8667 | 3560 | 41.08% |
| Other | 3183 | 1274 | 40.03% |
| Asian | 6133 | 1201 | 19.58% |
| Islander | 129 | 8 | 6.20% |
| Native | 187 | 6 | 3.21% |

We further verify the average income for different races in our data set. The distribution is quite consistent as the poverty rate data. Asian and Islander (Native Hawaiian or Other Pacific)’s average income is higher than White’s. Native’s (American Indian or Alaska Native) average income is a little lower than White’s, but close.

Table A8. Average Income of Each Race

| **Race** | **Average Income** |
| --- | --- |
| American Indian or Alaska Native | 70.0913 |
| Asian | 101.4268 |
| Black or African American | 57.6560 |
| Hispanic | 76.3149 |
| Multiracial | 67.8830 |
| Native Hawaiian or Other Pacific Islander | 103.4693 |
| Other | 84.7833 |
| Patient Refused | 72.7936 |
| Unknown | 66.6082 |
| White or Caucasian | 79.8272 |
| (blank) | 131.4640 |

Based on this finding, we add Asian, Native and Islander into the non-URM group, and rerun the analysis. Table A9 shows the updated results, which are qualitatively the same. Some effects are more significant.

Table A9. Effect of COVID on the Digital Divide with updated URM categorization

| **DV: ln (Usage)** | **OLS** | **Random Effect** | **Fixed Effect** |
| --- | --- | --- | --- |
| COVID_Period | 0.263 (0.004) | 0.253 (0.003) | 0.251 (0.003) |
|  | *P <* 0.001 | *P <* 0.001 | *P <* 0.001 |
| Minority | -0.125 (0.006) | -0.119 (0.011) |  |
|  | *P <* 0.001 | *P <* 0.001 |  |
| COVID_Period ´ Minority | 0.018 (0.009) | 0.021 (0.007) | 0.022 (0.007) |
|  | *P =* 0.049 | *P =* 0.004 | *P =* 0.002 |
| Age | -0.002 (0.0002) | -0.002 (0.0003) |  |
|  | *P <* 0.001 | *P <* 0.001 |  |
| Male | -0.056 (0.004) | -0.055 (0.008) |  |
|  | *P <* 0.001 | *P <* 0.001 |  |
| BMI | 0.006 (0.0003) | 0.006 (0.001) |  |
|  | *P <* 0.001 | *P <* 0.001 |  |
| Abnormal BP | -0.008 (0.004) | -0.011 (0.008) |  |
|  | *P =* 0.059 | *P =* 0.210 |  |
| COVID problem | 0.218 (0.032) | 0.257 (0.069) |  |
|  | *P <* 0.001 | *P <* 0.001 |  |
| Office visits | 0.348 (0.003) | 0.210 (0.002) | 0.172 (0.002) |
|  | *P <* 0.001 | *P <* 0.001 | *P <* 0.001 |
| Income | -0.0001 (0.00004) | -0.0001 (0.0001) |  |
|  | *P =* 0.017 | *P =* 0.202 |  |
| Constant | 0.449 (0.015) | 0.478 (0.028) |  |
|  | *P <* 0.001 | *P <* 0.001 |  |
| Marital Status FE | Yes | Yes | -- |
| Insurance FE | Yes | Yes | -- |
| Month FE | Yes | Yes | Yes |
| Observations | 317,796 | 317,796 | 317,796 |
| R^2^ | 0.123 | 0.068 | 0.056 |

1. Source: <https://worldpopulationreview.com/us-cities/> [↑](#footnote-ref-1)
